# Transcriptome Sequencing of *Codonopsis pilosula* and Identification of Candidate Genes Involved in Polysaccharide Biosynthesis

**DOI:** 10.1371/journal.pone.0117342

**Published:** 2015-02-26

**Authors:** Jian Ping Gao, Dong Wang, Ling Ya Cao, Hai Feng Sun

**Affiliations:** 1 College of Pharmacy, Shanxi Medical University, Taiyuan, Shanxi, China; 2 College of Chemistry & Chemical Engineering, Shanxi University, Taiyuan, Shanxi, China; Nazarbayev University, KAZAKHSTAN

## Abstract

**Background:**

*Codonopsis pilosula* (Franch.) Nannf. is one of the most widely used medicinal plants. Although chemical and pharmacological studies have shown that codonopsis polysaccharides (CPPs) are bioactive compounds and that their composition is variable, their biosynthetic pathways remain largely unknown. Next-generation sequencing is an efficient and high-throughput technique that allows the identification of candidate genes involved in secondary metabolism.

**Principal Findings:**

To identify the components involved in CPP biosynthesis, a transcriptome library, prepared using root and other tissues, was assembled with the help of Illumina sequencing. A total of 9.2 Gb of clean nucleotides was obtained comprising 91,175,044 clean reads, 102,125 contigs, and 45,511 unigenes. After aligning the sequences to the public protein databases, 76.1% of the unigenes were annotated. Among these annotated unigenes, 26,189 were assigned to Gene Ontology categories, 11,415 to Clusters of Orthologous Groups, and 18,848 to Kyoto Encyclopedia of Genes and Genomes pathways. Analysis of abundance of transcripts in the library showed that genes, including those encoding metallothionein, aquaporin, and cysteine protease that are related to stress responses, were in the top list. Among genes involved in the biosynthesis of CPP, those responsible for the synthesis of UDP-L-arabinose and UDP-xylose were highly expressed.

**Significance:**

To our knowledge, this is the first study to provide a public transcriptome dataset prepared from *C*. *pilosula* and an outline of the biosynthetic pathway of polysaccharides in a medicinal plant. Identified candidate genes involved in CPP biosynthesis provide understanding of the biosynthesis and regulation of CPP at the molecular level.

## Introduction

Codonopsis Radix (CR, known as “Dangshen” in Chinese) is a famous traditional Chinese herbal medicine that has been widely used in Chinese medicine for replenishing qi (vital energy) deficiency, strengthening the immune system, improving poor gastrointestinal function, curing gastric ulcer and appetite, etc [1]. In Chinese Pharmacopoeia, CR is prescribed as dried roots of *Codonopsis pilosula* (Franch.) Nannf., *C*. *pilosula* Nannf. var. *modesta* (Nannf.) L. D. Shen and *C*. *tangshen* Oliv. of the family Campanulaceae [2]. Among these source plants, *C*. *pilosula* (Franch.) Nannf. is widely distributed in China and the raw herb from the species accounts for the largest part of the market[3]. At present, commercial CR is predominantly produced in Shanxi, Shaanxi, Gansu, and Sichuan. Among these different resources, CRs from Lingchuan, Shanxi taste sweet and have been considered to be of good quality for hundreds of years [4]. Chemical and pharmacological studies of CR have shown that codonopsis polysaccharides (CPPs) are major contributors to the sweet taste and are capable of modulating immune system functions, preventing tumor growth, improving memory, curing diabetes, and increasing hemoglobin [5–10]. These documents also reveal that the components’ bioactivities are related to their structure and composition. Because the content and composition of CPPs varies according to the geographic region, method used for cultivation, and the age of the plant, it is important to understand the biosynthesis, metabolism, and regulation of CPPs [11].

Using simple capillary electrophoresis, Yang et al. demonstrated that CPP is an acidic hetero-polysaccharide composed of arabinose, glucose, rhamnose, galactose, mannose, glucuronic acid, and galacturonic acid in the molar contents of 48.1, 103.5, 16.1, 48.5, 7.5, 4.2, and 119.1 μmol, respectively [12]. Structural analysis of CPPS(3), a water-soluble polysaccharide, has shown that it is composed of galactose, arabinose, and rhamnose in a molar ratio of 1.13:1.12:1 [13]. There is remarkable diversity in the composition and structure of CPPs [7, 14, 15]. However, the mechanism by which CPPs are produced and accumulated in *C*. *pilosula* is not known. Additionally, pathways responsible for polysaccharide biosynthesis in other medicinal plants are also poorly understood [16, 17]. Use of efficient high throughput techniques may allow the identification of genes involved in the biosynthesis of polysaccharides in medicinal plants.

Transcriptome sequencing using next-generation sequencing (NGS) technology provides much information within a short time and with enormous depth and coverage [18]. To date, a large volume of sequences relevant to the biosynthesis of a number of natural products has been identified using this technique [19–24]. However, these efforts focused primarily on the biosynthesis of flavonoids, unsaturated fatty acids, triterpenoid sapogenins, and saponins. In the current study, a combined transcriptome of roots, stems, leaves, and flowers of *C*. *pilosula* grown in Lingchuan was developed with the help of NGS. The sequences were then analyzed to understand the biosynthesis of secondary products, particularly to identify candidate genes involved in CPP biosynthesis. Finally, to verify the quality of the dataset as well as to characterize the biosynthesis of CPP, identified classical transcripts with respect to different branches of CPPs biosynthesis were cloned and their abundance quantified by real time PCR. Our results provide insight into the biosynthesis of polysaccharides in *C*. *pilosula* and provide new avenues for sustainable production of bioactive natural products such as CPPs.

## Materials and Methods

### Ethics statement

The study was carried out on a private land and Guo Feng Zhao should be contacted for future permissions. The location is not protected in any way, and the field studies did not involve endangered or protected species.

### Plant material

Two-year-old *C*. *pilosula* planted in a field, where good agricultural practices were implemented, was harvested and used as plant material in 2012 and 2013. The field was located in Lingchuan, Shanxi, China (1,522 m altitude, 35°47′N, 113°24′E).

**Fig 1:**
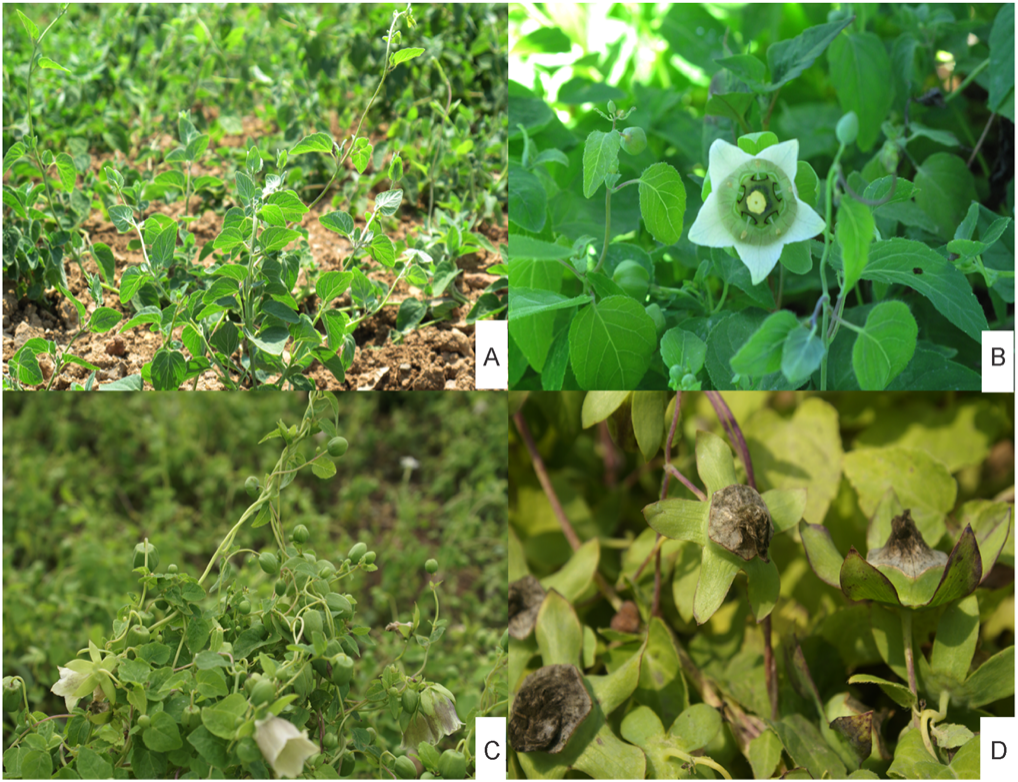
Plant material. Morphological characters of the aerial parts of 2-year-old plants at representative growth stages. (A) Tendril-pulling stage, stem elongated; (B) Flowering and boll-forming stage, stem extended further and the number of stalks and leaves went up sharply, flower buds become visible in appearance until petals extend; (C) Flowering stage, the number of flower buds increased and flowers open; (D) Seed maturity stage, seeds begin to be mature, and capsule hemispheric are at base, conical turns toward apex.

Previous studies have shown that the level of CPPs in root tissue of *C*. *pilosula* increases steadily during the growing season [25, 26]. At flowering and boll-forming stage (Fig. 1B), the content of CPPs was the highest in roots, and the content in flower buds was relatively higher than in other aerial parts (S1 Fig.). Thus, to comprehensively generate the *C*. *pilosula* transcriptome, flower buds, stems, leaves, as well as roots at this stage (Fig. 1B) were collected in the midafternoon of July 18, 2012. The other root materials were sampled in the midafternoon of June 18, August 8, and September 5 in 2012. At these sampling points, the plants were at the following growth stage of the year: tendril-pulling stage, flowering stage, seed maturity stage. Morphological characterization of the plants at each of these stages was presented in Fig. 1. After collection, the specimens were first washed with tap water, dried using filter paper, and then quickly cut into small pieces on ice before being snap-frozen in liquid nitrogen. The samples were stored at -70°C for library preparation and RNA-sequencing (RNA-seq).

For real time PCR analysis, leaves, stems, flower buds, and roots were collected in the midafternoon of July 27, 2013, at the flowering and boll-forming stage of plants. They were sampled, washed, and stored using the same method as that described above.

### Library preparation and sequencing (mRNA-seq)

Total RNA was isolated from each sample using a TRIzon kit (CWBIO, Beijing, China) following the manufacturer’s instructions. DNA contamination was removed using a Qiagen RNase free DNase I (Qiagen, Hilden, Germany). The integrity of isolated RNA was verified by agarose gel electrophoresis. RNA was quantified using a NanoDrop 2000 spectrophotometer (Thermo Fisher Scientific Inc., Wilmington, USA). Equal quantities of high-quality RNA from each sample were pooled and used for cDNA synthesis.

The cDNA library was constructed using a mRNA-Seq Sample Preparation Kit (Illumina Inc., San Diego, USA) according to the manufacturer’s recommendations. Briefly, mRNA was purified from the total RNA by using mRNA-Seq Sample Preparation Kit. The purified mRNA was then fragmented into small pieces by using fragmentation buffer and the first strand cDNA was synthesized from fragmented mRNA by using random hexamers as primers. After the synthesis of the second strand cDNA using DNA polymerase I (Invitrogen, Carlsbad, CA, USA) and RNase H (New England Biolabs Inc., Ipswich, MA, USA), a 3′-adenine was added to the terminus for further ligation of Solexa adapters. Ligation products were separated on a 2% TAE agarose gel. A specific region (200 ± 25 bp) of the gel was excised and the cDNA contained in the excised gel was purified using a gel extraction kit (Qiagen). These fragments were used for 12 rounds of PCR amplification with primers complementary to the previously ligated adaptors. The final mRNA-seq libraries were constructed after the products were purified using a QIAquick PCR kit (Qiagen). The mRNA-seq libraries were sequenced on an Illumina Genome HiSeq 2000 platform (BioMarker Technologies Co., Ltd, Beijing, China).

### *De novo* assembly and functional annotation

Clean reads were obtained by removing the adapter and low quality sequences as well as sequences that were less than 60 bases in length. *De novo* assembly was carried out using Trinity [27]. The K-mer value was set at 25 while other parameters were set at default levels. Redundancy removal was further performed with sequence clustering software TGICL to acquire non-redundant unigenes [28]. The unigenes obtained after gene family clustering were composed of clusters (corresponding prefix was CL), which contained several unigenes and singletons (the prefix was Unigene). Similarity between unigenes within one cluster was higher than 70%.

The unigenes thus obtained were aligned (online) to the following databases: GenBank non-redundant (NR, http://www.ncbi.nlm.nih.gov), Swiss-Prot (http://web.expasy.org/docs/relnotes/relstat.html), COG (http://www.ncbi.nlm.nih.gov), GO (http://www.geneontology.org/), and KEGG (http://www.genome.jp/kegg/) [29–31]. A cut-off value of E < 10^-5^ was set for all BLASTx searches. Based on BLASTx hits against the NR database, BLAST2GO program was carried out to obtain the GO annotations [32]. WEGO was used to obtain GO classifications according to molecular function, biological process, and cellular component [33].

### Prediction of the protein-coding region

For predicting the protein-coding region, the unigenes were first aligned (E-value <10^-5^) to protein databases in the priority order of NR, Swiss-Prot, KEGG, and COG. The unigenes aligned to a database of higher priority were no longer aligned to a lower priority database. Proteins in the blast results with the highest ranks were selected and the coding sequences (CDSs) as well as the amino acid sequences were determined using standard codons as references. Unigenes that did not align to any available database were scanned using ESTScan, which produced nucleotide and amino acid sequences of the predicted coding region [34].

### Analysis of the genes involved in the biosynthesis of CPPs

The analysis of genes involved in the biosynthesis of CPPs depended on the KEGG pathway annotation dataset. Candidate genes involved in CPPs biosynthesis were identified in KEGG database based on the metabolic pathways of starch, glucose, amino sugar, and nucleotide sugar. Neighbor joining (NJ) phylogenetic analysis of *UGPase* (uridine diphosphate glucose pyrophosphorylase) was performed with MEGA5[35]. All potential transcripts annotated as *GT*s (glycosyltransferases) [EC: 2.4.x.y] were extracted with the help of Perl script based on the Swiss-Prot annotation and were classified by BLASTx searches against local *Arabidopsis thaliana* GTs database using a threshold of 1.0^-5^, which encompasses all genes encoding GTs in *A*. *thaliana* (http://www.cazy.org/e1.html).

### Real-time PCR

Nine genes potentially involved in the biosynthesis of CPPs were selected for further analysis. Specific primers were designed using Primer Premier 3.0 [36]. The details regarding the genes and primers are presented in Table 1. For expression analysis, total RNA was extracted from the leaf, stem, flower bud, and root tissues at the same growth stage in 2013 using the protocol described earlier. The RNA was treated with DNase I to remove contaminating DNA (TaKaRa, Dalian, China). Following this, total RNA was reverse transcribed into cDNA by using a PrimeScript RT Master Mix (TaKaRa). All primer pairs were examined using standard real-time PCR and Premix Ex Taq (TaKaRa), and the presence of a single amplification product of the expected size for each gene was verified by electrophoresis on a 1.5% agarose gel followed by ethidium bromide staining.

**Table 1:** *Codonopsis pilosula* candidate genes and primers used in real-time PCR.

Gene ID	Candidate gene	Upstream primer (5′-3′)	Tm(°C)	Downstream Primer (5′-3′)	Tm(°C)	Length(bp)
Unigene12384	*GAPDH*	tgcttcgttcaacatcattc	58.1	cataactggctgccttctcc	61.9	164
Unigene186	*manA*	tttgcggttactattcactc	56.9	acatcatctggatactgctt	57.4	162
Unigene11590	*manB*	aacgccaactgagacaac	59.4	gcactcttacagcaccga	60.9	86
Unigene16117	*UGPase*	tttacccttgagaacgacg	58.6	tctgatggctatgtgaccc	60.1	192
Unigene7955	*RHM*	ctgactcttgctgatgttt	53.2	gaatccaataccagaccct	55.4	111
Unigene12223	*UER*	ccaatccccgtaacttcat	58.3	tattccagtcaggttcctcttt	59.8	131
CL5269.Contig2	*UGDH*	gatgcttatgaggcgacgaa	61.6	gaggcttaccaatggagtagacaat	63.2	193
Unigene9227	*UXE*	tgttggcacaggaagaggt	62.6	ccgacgaggaaggaaatca	60.6	97
Unigene15345	*UGlcAE*	cacgggattttacctacat	55.7	cttcttcttaccgcctga	57.3	95
Unigene14947	*AXS*	gataaaggcgatgacgata	55.7	aagacggttcaagaaggtg	58.8	146

Abbreviations: *GAPDH*—glyceraldehyde-3-phosphate dehydrogenase, m*anA*—mannose-6-phosphate isomerase, *manB*—phosphomannomutase, *UGPase*—uridine diphosphate glucose pyrophosphorylase, *RHM*—UDP-glucose 4,6-dehydratase, *UER*—3,5-epimerase/4-reductase, *UGDH*—UDP-glucose 6-dehydrogenase, *UXE*—UDP-arabinose 4-epimerase, *UGlcAE*—UDP-glucuronate 4-epimerase, *AXS*—UDP-apiose/xylose synthase.

Real-time PCR was performed using a SYBR Premix ExTaq Kit (TaKaRa) on an Applied Biosystems 7300 Real-Time PCR system (Applied Biosystems, Foster City, CA, USA). The total volume of the reaction mixture was 20 μL, and it contained 2 μL cDNA, 10 μL SYBR Premix Ex Taq, 0.4 μL ROX Reference Dye II, and each primer at a concentration of 4 pmol. PCR conditions were as follows: 95°C for 30 s, followed by 40 cycles of 95°C for 5 s and 60°C for 31 s. The relative expression levels of genes were calculated by 2^-ΔΔCt^ method [37, 38]. Biological samples from three independent experiments were analyzed and each reaction was run in triplicates. Raw data on the relative abundance of each transcript were expressed as mean ± standard deviation (SD).

## Results

### Illumina sequencing, *de novo* assembly, and assessment of assembly program

**Fig 2:**
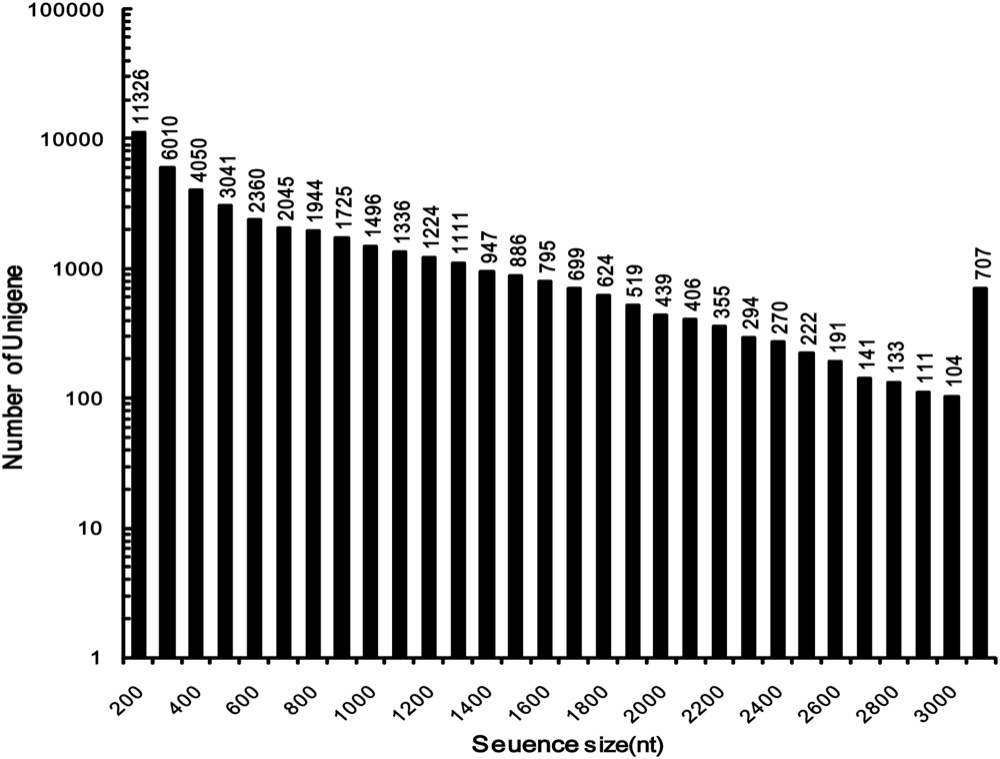
Length distribution of *Codonopsis pilosula* unigenes.

At the flowering and boll-forming stage, reproduction and vegetative growth concur in the species, and flower buds become gradually visible and the petals become extended (Fig. 1B). At this stage, concentrations of CPPs in the roots and flower buds are rather higher than in the other aerial parts. To cover the *C*. *pilosula* transcriptome comprehensively, total RNA was isolated from the four tissues collected at this stage as well as from the root tissue collected at other growth stages. Equal amounts of total RNA from these samples were collected and pooled. The mRNA was purified, fragmented, and reverse-transcribed into cDNA. The cDNA was subjected to Illumina HiSeq 2000 paired-end sequencing. After the removal of adaptor sequences, ambiguous reads, and low quality reads, 91,175,044 clean reads 101 bp in length, were obtained. All HiSeq 2000 sequencing data were combined and assembled using Trinity, which produced 102,125 contigs with an N50 of 999 bp (i.e., 50% of the assembled bases were incorporated into contigs of 999 bp or longer). Then, TGICL was used to reduce the redundancy. Finally, 45,511 unigenes were generated, which included 10,563 clusters and 34,948 singletons (Table 2). The size distribution of the unigenes is shown in Fig. 2. The size distribution was homogeneous and 25.3% of unigenes were longer than 1000 bp. The longest unigene recovered in this work was Unigene CL5433.Contig2_Codonopsis, the length of which was 9,150 bp and it encoded *alpha*, *alpha*-trehalose phosphate synthase (according to Swiss-Port annotation result). All reads were deposited in the National Center for Biotechnology Information (NCBI) and can be accessed in the Short Read Archive (SRA) Sequence Database under project accession number: SRP038022.

**Table 2:** Summary statistics of transcriptome library for *Codonopsis pilosula*.

Clean reads	91,175,044
Total nucleotides (bp)	9,208,077,400
Q20 percentage	92.1
GC percentage	46.91
Total number of contigs	102,125
Mean length of contigs (bp)	350
N50 length of contigs (bp)	999
Total number of Unigenes	45,511
Mean length of Unigenes (bp)	728
N50 length of Unigenes (bp)	1,243
Number of clusters	10,563
Number of singletons	34,948

Q20 means that the sequencing error rate was less than 1%. N50 is calculated by sorting all contigs or unigenes from the smallest to the largest and determining the size at which 50% of all bases in the assembly are contained in contigs or unigenes.

### Functional annotation and analysis of the predominant transcripts

All *C*. *pilosula* unigenes generated by HiSeq 2000 sequencing were aligned to public protein databases (NR, COG, Swiss-Prot, and KEGG) and nucleotide database (Nt) by BLASTx and BLASTn, respectively (with cut-off E-values of 10^-5^). In total, 34,616 (76.1%) unigenes were aligned to homologous sequences in public databases (Table 3). To understand the expression profile of the assembled sequences, we aligned the high-quality clean reads to the assembled *C*. *pilosula* transcripts with the help of Bowtie program, allowing one mismatch. Following the alignments, the count of raw reads for each unigene was normalized to fragments per kilobase of exon model per million mapped fragments (FPKM) [39]. We also mapped the reads to the assembled transcriptome; 92.51% of the reads could be mapped to the *C*. *pilosula* library. The data on the mapped efficiency are presented in S1 Table.

**Table 3:** Summary statistics of functional annotations for *Codonopsis pilosula* unigenes in public databases.

Annotated databases	Number of Unigenes	Percentage (%)
NR	33,748	74.2
Nt	27,319	60.0
GO	26,189	57.5
COG	11,415	25.1
KEGG	18,848	41.4
Swiss-Prot	21,265	46.7
Total	34,616	76.1

### NR annotation

**Fig 3:**
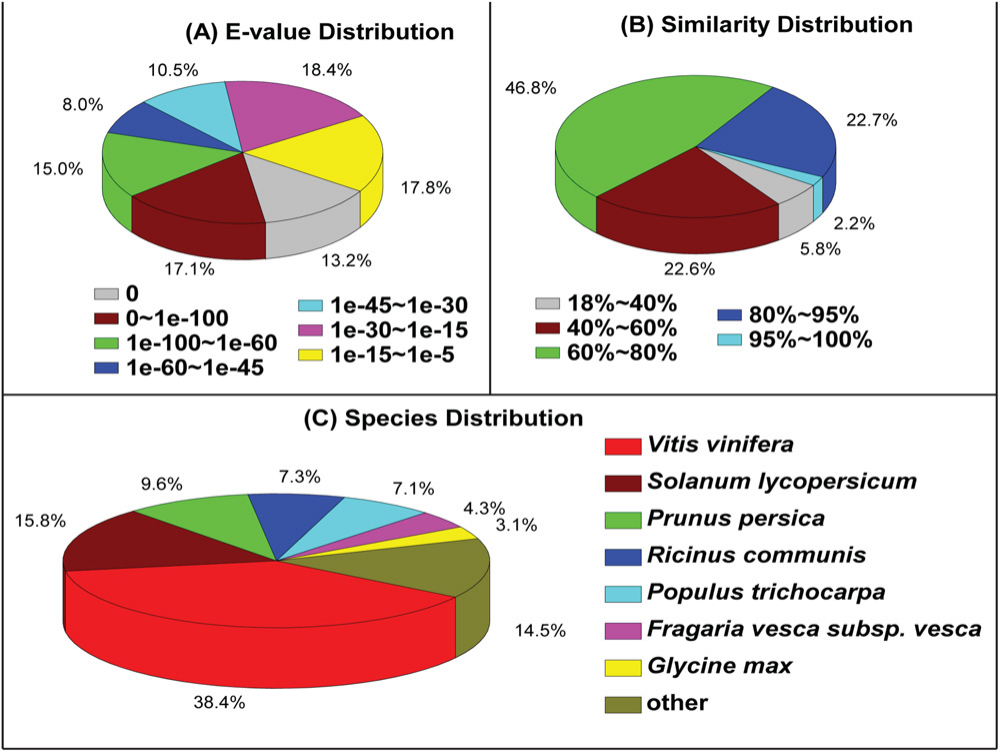
Characteristics of the alignment of *Codonopsis pilosula* unigenes against the NR database. (A) E-value distribution of the top BLAST hits for each Unigene. (B) Similarity distribution of the top BLAST hits for each unigene. (C) Species distribution of the top BLAST hits for all homologous sequences.

Approximately 74.2% (33,748) of the unigenes were annotated with reference to the NR database. Based on the NR annotations, 30.3% of the annotated sequences had the highest homology (E-value < 10^-100^) and 15% had moderate homology (10^-100^ < E-value < 10^-60^) (Fig. 3A). With respect to similarity distribution, 24.9% of the hits had higher similarity than 80%, and 46.8% had moderate similarities of approximately 60–80% (Fig. 3B). With respect to species, 38.4% of the unigenes had top matches to the sequences from *Vitis vinifera*, 15.8% to the sequences from *Solanum lycopersicum*, and 9.6% to those from *Prunus persica* (Fig. 3C).

### GO categories

**Fig 4:**
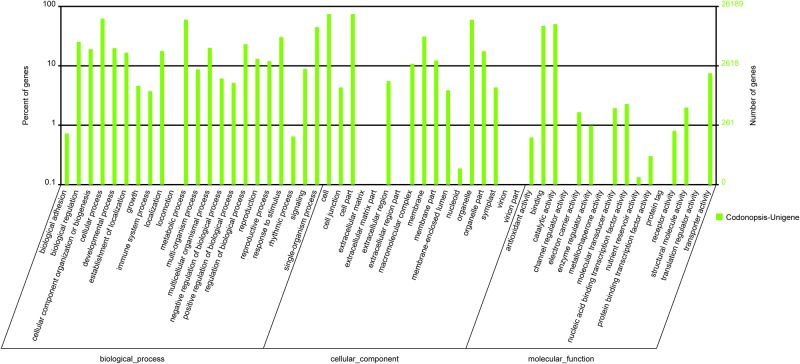
GO classification of the unigenes derived from *Codonopsis pilosula*. The histogram showed the result of classifying 26,189 genes into the secondary classification of GO terms. Right Y-axis: number of genes; Left Y-axis: percentage of genes.

Among the 33,748 most significant BLASTx hits against the NR database, 26,189 unigenes could be assigned to one or more terms, which were categorized into 55 functional groups as shown in Fig. 4. Among the unigenes that were categorized in “the biological process,” 16,286 were related to the “cellular process” and represented the largest proportion, followed by “metabolic process” (15,732), and “single-organism process” (11,743). Within the cellular components, the assignments were mostly enriched in the “cell part” (19,471) and “cell” (19,472). The GO terms for molecular function were mainly grouped into “catalytic activity” (13,164) and “binding” (12,307).

### COG annotation

**Fig 5:**
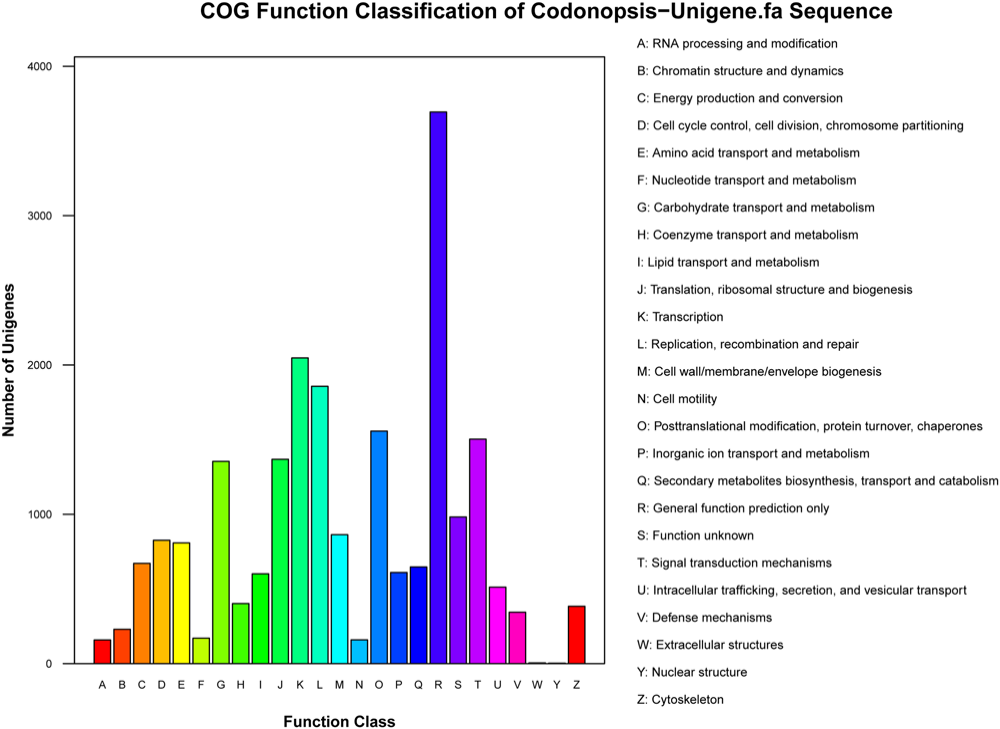
COG functional classification of *Codonopsis pilosula* transcriptome.

Assignment of COG was used to further evaluate the completeness of *C*. *pilosula* transcriptome library and the reliability of the annotation process. Overall, 11,415 (25.1%) unigenes were assigned to 25 COG categories (Fig. 5). Among these groups, unigenes belonging to the “general function prediction” occupied the largest part (3,694, 32.6%), followed by “transcription” (2,047, 17.9%), “replication, recombination, and repair” (1,857, 13.8%). Additionally, 1,354 (11.7%) unigenes were assigned to “carbohydrate transport and metabolism,” and 647 (5.7%) were assigned to “secondary metabolites biosynthesis, transport, and catabolism.” Unigenes assigned to “extracellular structures” and “nuclear structure” constituted the smallest category.

### KEGG pathway mapping

**Fig 6:**
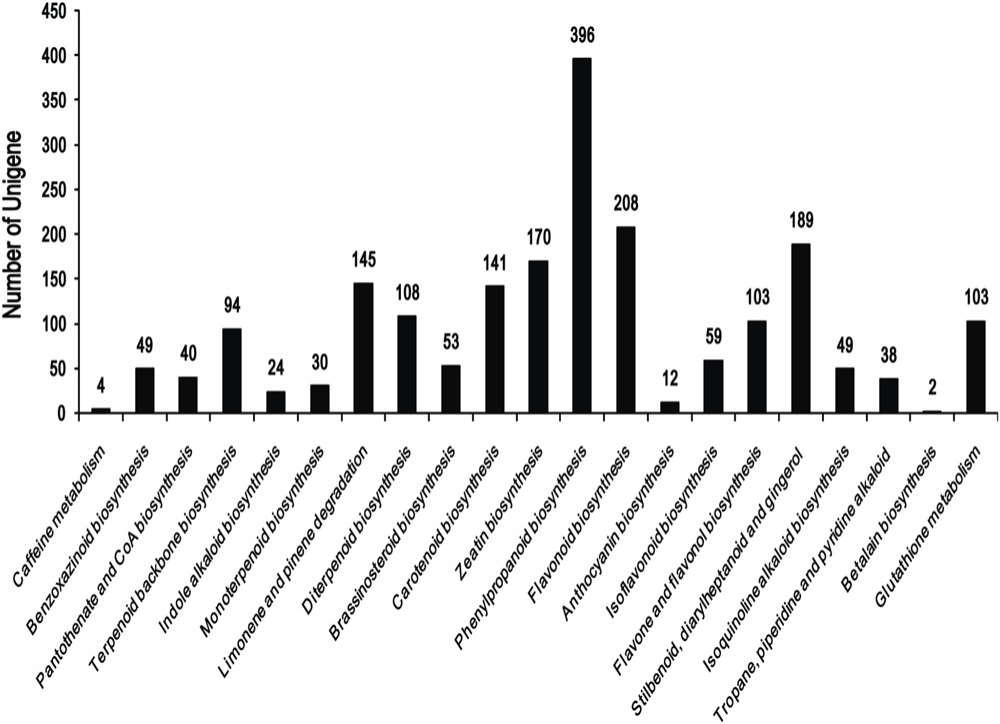
Unigenes from *Codonopsis pilosula* assigned to secondary metabolic pathways.

The unigenes annotated to the KEGG database were mapped to further understand the biological processes in *C*. *pilosula*. Totally, 18,848 unigenes were assigned to 128 KEGG pathways (see S2 Table). Among these, 4,191 (22.24%) were mapped to metabolic pathways and 2,076 (11.01%) were mapped to biosynthesis of secondary metabolites (Fig. 6). Moderate numbers of unigenes were mapped to the following pathways: “plant-pathogen interaction” (1,096, 5.81%), “plant hormone signal transduction” (986, 5.23%), “RNA transport” (747, 3.96%), “spliceosome” (601, 3.19%), “starch and sucrose metabolism” (582, 3.09%), “glycerophospholipid metabolism” (506, 2.68%), and “endocytosis” (501, 2.66%). Only four unigenes were distributed to “betalain biosynthesis” and two unigenes were distributed to “Caffeine metabolism” in the library.

### Analysis of highly expressed transcripts

The value of FPKM represents the abundance of a certain unigene in a certain gene pool, and is often used to indicate the level of gene expression in various biological samples. The top 20 relatively highly abundant genes in the dataset are listed in Table 4. Transcripts encoding chlorophyll a-b binding protein 40, ribulose-1,5-bisphosphate carboxylase small subunit, and chloroplast photosystem II 10 kDa polypeptide were abundant in the transcriptome library, indicating that the photosynthetic process was active in the tissues investigated. Metallothionein is responsible for the detoxification of heavy metals, metal ion transport, maintenance of dynamic balance, ion metabolism, and reactive oxygen removal [40]. Two transcripts (Unigene8032_Codonopsis and Unigene12118_Codonopsis) encoding metallothionein were also found abundantly in the tissues. Additionally, unigenes encoding aquaporin, cysteine protease, chloroplastic thiamine thiazole synthase, and dormancy-associated protein were also abundant in the cDNA library. The remaining unigenes with higher FPKM values could not be annotated successfully, indicating their unidentified roles in *C*. *pilosula*.

**Table 4:** Predominant unigenes in *Codonopsis pilosula* transcriptome library.

Gene ID	FPKM	NR-ID	NR-annotation
CL3472.Contig1	41524.464	ref|XP_003614394.1|	Hypothetical protein
Unigene9157_Codonopsis	33994.62	ref|XP_003614382.1|	Hypothetical protein
CL595.Contig1_Codonopsis	6054.2321	ref|XP_003614391.1|	Tar1p
CL655.Contig2_Codonopsis	4182.2431	ref|XP_002285299.1|	RD21a-like cysteine proteinase
Unigene8032_Codonopsis	3955.4621	dbj|BAD18924.1|	Metallothionein 2
Unigene14155_Codonopsis	3759.3688	ref|XP_004170751.1|	Uncharacterized protein
Unigene17719_Codonopsis	2440.7379	ref|XP_002510636.1|	Phosphoprotein ECPP44
CL40.Contig5_Codonopsis	2340.6985	sp|P27495.1|CB24_TOBAC	Chlorophyll a-b binding protein 40
Unigene9176_Codonopsis	2271.6946	gb|AAA33866.1|	Ribulose-1,5-bisphosphate carboxylase small subunit
CL2635.Contig3_Codonopsis	1994.5632	gb|ACB38232.1|	Aquaporin
Unigene12118_Codonopsis	1818.9061	gb|AAO92264.1|	Metallothionein-like protein
Unigene3690_Codonopsis	1786.4363	gb|ADB93062.1|	Chloroplast photosystem II 10 kDa polypeptide
CL655.Contig1_Codonopsis	1759.3512	gb|AAW34135.1|	Cysteine protease gp2b
Unigene3692_Codonopsis	1680.0371	gb|EMJ23383.1|	Hypothetical protein
Unigene3701_Codonopsis	1610.1937	ref|XP_002301827.1|	Predicted protein
CL655.Contig3_Codonopsis	1584.7255	sp|P60994.1|ERVB_TABDI	Ervatamin-B
Unigene15471_Codonopsis	1543.9714	sp|O23787.1|THI4_CITSI	Thiamine thiazole synthase, chloroplastic
Unigene12117_Codonopsis	1407.0655	ref|XP_002513211.1|	Lipid binding protein
Unigene9175_Codonopsis	1370.3016	gb|AAW02792.1|	Dormancy-associated protein

### Predicted coding sequences

**Fig 7:**
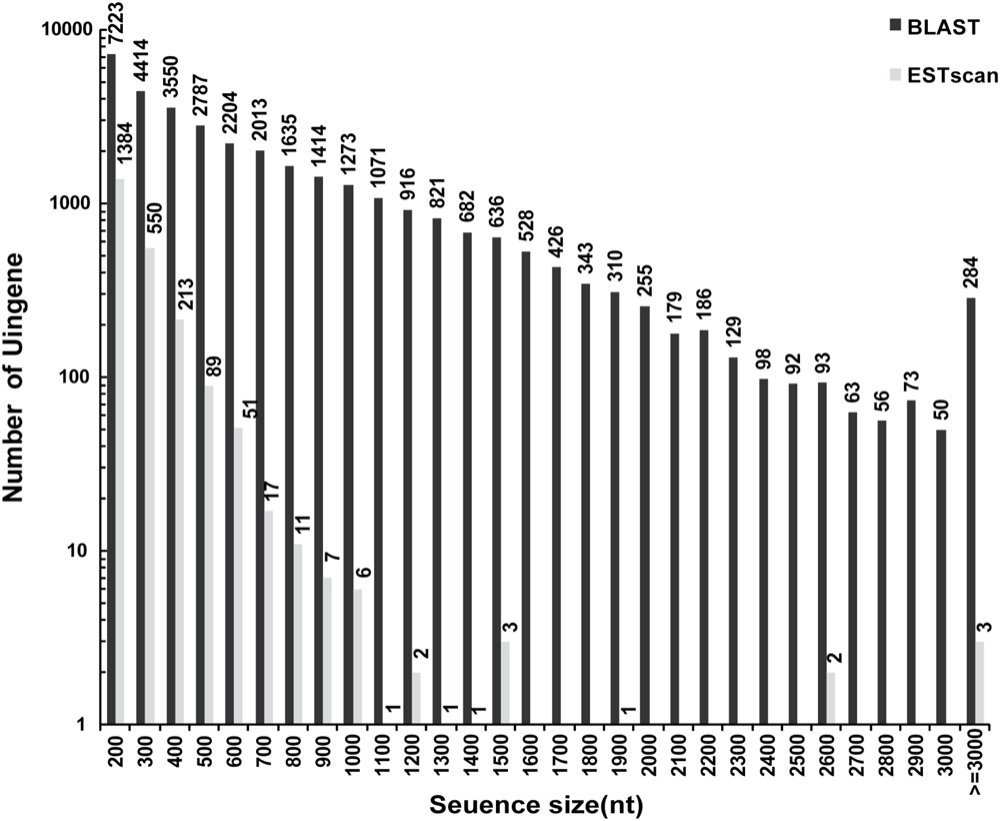
Characterization of the *Codonopsis pilosula* transcriptome. (A) Size distribution of CDSs when aligned with available protein databases (in the order of NR, SwissProt, KEGG, and COG) using BLASTx (E-value < 10^-5^); (B) Size distribution of proteins deduced from the CDSs. For unigene CDSs that had no hits in the available databases, the BLAST was subjected to ESTScan and translated to peptide sequences.

Based on three public protein databases, 36,146 CDSs were obtained, which accounted for 79.42% of the total number of unigenes. Among these, 33,804 CDSs were predicted by BLASTx and 2,342 by ESTScan, indicating relatively higher identities of the predicted CDSs to those available in the public databases. The length distribution of CDSs is shown in Fig. 7. A total of 27,562 CDSs were between 200 bp and 1000 bp in length, 68.25% of which was longer than 500 bp, thus constituting a relatively higher proportion of the predicted CDSs. The number of CDSs longer than 2000 bp was 1,563, which was approximately 18.21% of the CDSs longer than 1000 bp, indicating a relatively higher ratio of CDSs with larger size. The CDSs predicted by ESTScan tool were primarily 200 bp to 1000 bp in length.

### Functional annotation and candidate genes involved in CPP biosynthesis

To understand the biosynthesis of CPPs and to identify the genes involved, the transcripts related to starch and sucrose metabolism (ko00500) as well as amino sugar and nucleotide sugar metabolism (ko00520) were analyzed [41]. Intensive studies on cell wall polysaccharides have indicated that this fraction is variable in glycan structures and is mainly composed of xyloglucans, glucomannans, and pectic polymers. Across known cases the plant polysaccharides are constitutive of nucleotide-diphospho-sugar (NDP-sugar) precursors. A class of enzymes called glycosyltransferases (GTs) catalyzes specific sugar incorporation from an NDP-sugar into a growing polysaccharide chain. The composition of cell walls is determined in part by the availability of NDP-sugars to form different types of wall polymers [42–44]. Since all wall polysaccharides are synthesized from activated NDP-sugars, biosynthetic pathway of CPPs was inferred and predicted.

### Starch and sucrose metabolism

Among 128 metabolic pathways detected in *C*. *pilosula*, candidate genes related to starch and sucrose metabolic pathways were ranked seventh as far as the number of involved genes was concerned (S3 Table). To understand the biosynthesis of CPPs, the unigenes involved in the biosynthesis of starch and other related polymers as well as those related to carbon assimilation in the leaf tissues were annotated. The key enzymes involved in these metabolic processes were identified and annotated based on KEGG (see Table 5 and S3 Table).

**Table 5:** Unigenes involved in the biosynthesis of starch and sucrose in *Codonopsis pilosula*.

**Enzyme name**	**EC**	**Number of Unigenes**
Glucose-6-phosphate isomerase	5.3.1.9	2
Phosphoglucomutase	5.4.2.2	2
Uridine diphosphate glucose pyrophosphorylase	2.7.7.9	1
Cellulose synthase	2.4.1.12	55
Sucrose synthase	2.4.1.13	15
Sucrose-phosphate synthase	2.4.1.14	16
Glucose-1-phosphate adenylyltransferase	2.7.7.27	8
Starch synthase	2.4.1.21	13
Starch branching enzyme	2.4.1.18	13
Granule-bound starch synthase	2.4.1.242	1
Invertase	3.2.1.26	24
Hexokinase	2.7.1.1	7
Fructokinase	2.7.1.4	9

In plants, UGPase catalyzes the formation of uridine diphosphate glucose (UDP-Glu), a key precursor of NDP-sugars formed from 1-P-glucose (Glu-1-P) [45, 46]. In *Astragalus membranaceus*, the enzyme activity is positively correlated with the concentration of Astragalus polysaccharides, indicating that UGPase plays an important role in polysaccharide biosynthesis [16]. In *Arabidopsis thaliana*, UGPase is a rate-limiting factor and contributes primarily to UDP-Glu metabolism in the vegetative phase [45]. In *C*. *pilosula* library, Unigene16117_Codonopsis (KJ470627) was identified as a homologue of *UGPase*. This 2087-bp unigene contained a complete open reading frame (ORF) and showed the highest amino acid identity (88%) to UGPase from *V*. *vinifera* (Accession number: XP_002282276)(S2 Fig.). In *A*. *membranaceus*, granule-bound starch synthase (GBSS) is a key enzyme required for the synthesis of polysaccharides [17]. A 2308-bp Unigene7794_Codonopsis (KJ470628) was also identified as a homologue of *GBSS* and contained an intact ORF encoding 622 amino acids.

### Amino sugar and nucleotide sugar metabolism

Previous studies indicated that CPPs are formed by the polymerization of NDP-sugars such as UDP-glucose (UDP-Glu), UDP-glucuronic acid (UDP-GluA), UDP-arabinose (UDP-Ara), UDP-rhamnose (UDP-Rha), GDP-mannose (GDP-Man), UDP-galactose (UDP-Gla), UDP-xylose (UDP-Xyl), and UDP-galacturonic acid (UDP-GlaA) [12–15]. Among these NDP-sugars, UDP-Glu and GDP-Man are primarily derived from glucose-6-phosphate (Glu-6-P) and fructose-6-phosphate (Fru-6-P), whereas others are derived from UDP-Glu and GDP-Man through the actions of NDP-sugar interconversion enzymes (NSEs) such as isomerase, decarboxylase, and dehydrogenase [47]. Six subclasses of NSEs were identified in *C*. *pilosula*, including 4-epimerase, 3,5-epimerase, 3,5-epimer-4-reductase, 4,6-dehydrogenase, and 6-dehydrogenaseand decarboxylase.

Thirty-seven transcripts encoding NSEs were identified in *C*. *pilosula* (Table 6, S4 Table). A comparison of FPKM values revealed that in the transcriptome library, the transcript of *UGlcAE* (UDP-glucuronate 4-epimerase) was the most abundant, followed by those of *UGDH* (UDP-glucose 6-dehydrogenase) and *AXS* (UDP-apiose/xylose synthase). The protein encoded by *UGlcAE* is responsible for the production of UDP-GalA, using UDP-GluA as a precursor. The enzyme encoded by *UGDH* converts UDP-Glu into UDP-GluA, thus taking part in the biosynthesis of glycosaminoglycans. AXS converts UDP-GluA to UDP-Xyl. Relatively higher abundance of such transcripts suggested that the biological process of conversion from UDP-GluA to UDP-Xyl should be very active.

**Table 6:** Number of unigenes annotated as NDP-sugar interconversion enzymes (NSEs).

**Enzyme code**	**Enzyme name**	**Abbreviation**	**Number**	**FPKM**
**5.3.1.8**	mannose-6-phosphate isomerase	*manA*	2	19.3816
**5.3.1.9**	glucose-6-phosphate isomerase	*GPI*	2	158.1845
**5.4.2.8**	Phosphomannomutase	*manB*	5	111.3943
**2.7.7.13**	mannose-1-phosphate guanylyltransferase	*GMPP*	7	86.7738
**5.4.2.2**	Phosphoglucomutase	*pgm*	2	143.3946
**2.7.7.9**	Uridine diphosphate glucose pyrophosphorylase	*UGPase*	1	242.5288
**4.2.1.76**	UDP-glucose 4,6-dehydratase	*RHM*	4	222.6735
**5.1.3.-1.1.1.-**	3,5-epimerase/4-reductase	*UER*	1	110.7206
**5.1.3.6**	UDP-glucuronate 4-epimerase	*UGlcAE*	4	488.2218
**-**	UDP-apiose/xylose synthase	*AXS*	1	343.0033
**5.1.3.5**	UDP-arabinose 4-epimerase	*UXE*	1	48.15
**5.1.3.2**	UDP-glucose 4-epimerase	*UGE*	2	125.0329
**5.1.3.18**	GDP-mannose 3,5-epimerase	*GME*	1	18.1922
**1.1.1.22**	UDP-glucose 6-dehydrogenase	*UGDH*	4	400.1352

### CPP biosynthetic pathway

**Fig 8:**
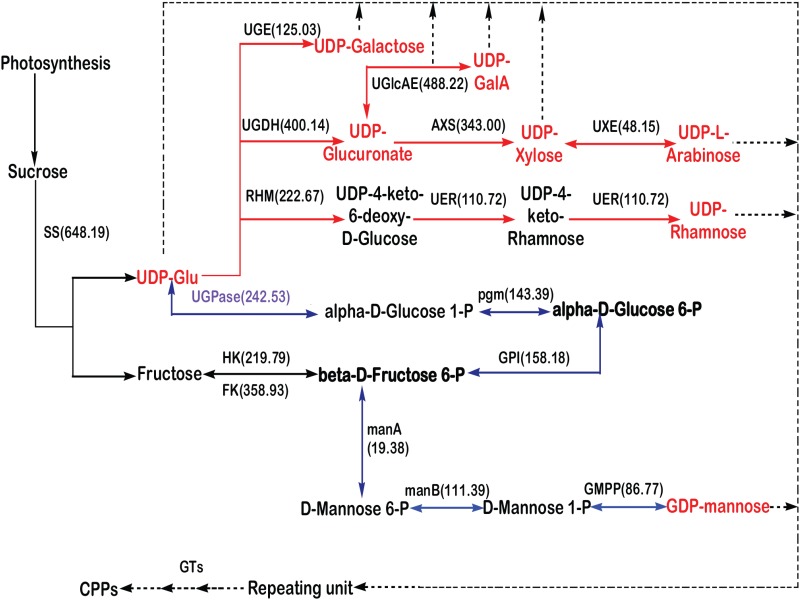
Proposed pathways for polysaccharide biosynthesis in *Codonopsis pilosula*. Activated monosaccharide units were marked in red. Words in bold are key intermediates. The first step of CPPs biosynthesis was shown with blue arrows. Conversion of monosaccharides were marked with red arrows. The key enzymes were highlighted in purple, and the corresponding FPKM values were indicated in brackets. Abbreviations: SS: sucrose synthase; HK: fructokinase/hexokinase; UGPase: uridine diphosphate glucose pyrophosphorylase; RHM: UDP-glucose 4, 6-dehydratase; UGDH: UDP-glucose 6-dehydrogenase; UGE: UDP-glucose 4-epimerase; UGlcAE: UDP-glucuronate 4-epimerase; AXS: UDP-apiose/xylose synthase; UXE: UDP-arabinose 4-epimerase; UER: 3, 5-epimerase/4-reductase; PGM: phosphoglucomutase; GPI: glucose-6-phosphate isomerase; manA: mannose-6-phosphate isomerase; manB: phosphomannomutase; GMPP: mannose-1-phosphate guanylyltransferase; GTs: glycosyltransferases.

Based on the annotation results of the abovementioned pathway and documents published[41], biosynthetic pathway for the formation of CPPs from sucrose was cautiously constructed and was shown in Fig. 8. In this scheme, synthesis of CPPs is divided into three main steps. The first step is the formation of UDP-Glu and GDP-Man from Glu-6-P and Fru-6-P, which are then converted in the second step into other NDP-sugars. Finally, the active monosaccharide units, NDP-sugars, are added to the sugar residues of various polysaccharides and glycoconjugates by the action of various GTs [48].

In *C*. *pilosula* library, 723 transcripts encoding GTs were identified based on the annotations in *Arabidopsis thaliana*, including 591 GTs and 132 GTNCs (referenced for glycosyltransferases with no classification) (see S5 Table). Among these *GTs*, the transcripts of family 1 glycosyltransferase (GT1s), often referred to as UDP glycosyltransferase (UGTs), were the most abundant, indicating that GT1s might be encoded by comparably large multigene families. The transcripts of *GT2*, *GT8*, *GT4*, and *GT48* were more abundant than that of others, and *GT31*, *GT20*, *GT41*, *GT5*, and *GT90* showed moderate abundance, whereas the remaining had only limited number of transcripts (less than ten in number). An imbalance in the number of transcripts was also observed in annotated *UGTs*. Since these genes have specific functions in the biosynthesis of CPPs, the differing abundances are indicative of a preference for the synthesis of specific NDP-sugars.

In addition to polysaccharides, UGTs are also responsible for the transfer of sugar moieties from UDP sugars to other plant natural products, such as phenolic compounds, cyanins, steroids, flavonoids, and terpenes [49]. In general, approximately 48% of UGTs contain a common motif known as plant secondary product glycosyltransferase (PSPG) box [50]. As key sugar donors, NDP-sugars are primarily attached to PSPG motif of UGTs. Therefore, the PSPG motif is very important for UGTs. This motif contains 44 conserved amino acids. The amino acids at C-terminus are glutamine (Q) and histidine (H), which are highly conserved in glycosyltransferase and galactosyltransferase respectively [50]. An analysis of the amino acid sequences of PSPG in the annotated UGTs of *C*. *pilosula* showed that the terminal amino acid residue of all annotated UGTs was Q except in unigene2366, whose terminal amino acid residue was H, indicating an obvious imbalance in the transfer of NDP-sugars.

**Fig 9:**
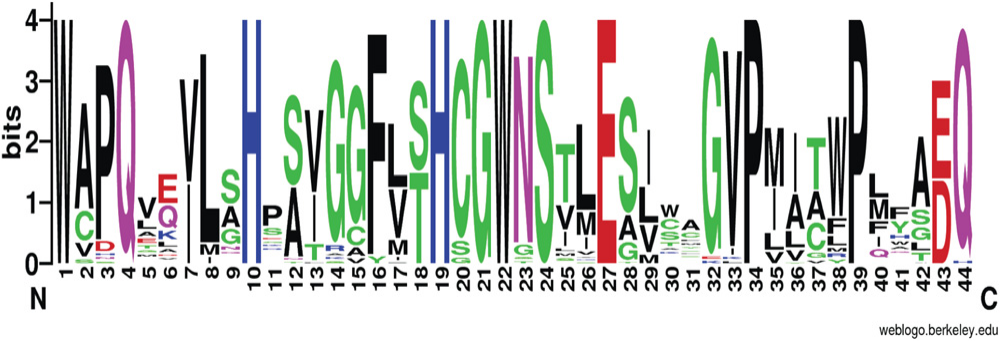
Consensus sequence defining the glycosyltransferase PSPG motif in plants. The figure was generated using WebLogo (http://weblogo.berkeley.edu/logo.cgi). Amino acid consensus sequences of UGTs from *C*. *pilosula* refer to the PROSITE PS00375. The font size is indicative of the degree to which the amino acid residues are conserved (larger font size indicates more conserved residues).

Amino acid sequences after multiple sequence alignments reflect their PSPG structures and their functions. Therefore, amino acid sequence analysis of this motif will provide insights into their functional diversities in CPPs biosynthesis. Conservative analysis on the amino acid sequences of fifty UGTs possessing a typical PSPG sequence was performed using WebLogo (http://weblogo.berkeley.edu/logo.cgi) and is presented in Fig. 9. The font size represented the conservative properties of amino acid residues, the bigger the font the more conservative amino acid residue. It indicates that the first and twenty-second tryptophan (W), fourth and forty-fourth glutamine (Q), tenth and nineteenth histidine (H), twenty-first glycine (G), twenty-fourth serine (S), twenty-seventh glutamic acid (E), and thirty-fourth and thirty-ninth proline (P) residues at N-terminus are more conserved than other amino acids in the motif. Studies on the structure-activity relationship of UGT have demonstrated that the nucleotide sugar donor primarily interacts with the C-terminal domains and that the acceptor mainly binds to the N-terminal domains [50]. Since diverse natural products act as acceptors of UGTs, further studies are needed to understand whether specific UGTs are involved in CPPs biosynthesis.

### Real-time PCR analysis

Analysis of the abundance of transcripts of enzymes related to CPP biosynthesis provides evidence at two levels. On one hand, the detection of PCR products verifies if the sequences obtained by high throughput screening are reliable and accurate. On the other hand, it provides insights into their functions in different branches of CPPs biosynthesis. Based on the preliminary results of the selection of reference genes in *C*. *pilosula* (see S6 Table), *GAPDH* was used as internal reference in the present study. Nine candidate unigenes were selected based on the alignment results from online BLAST for verification. Among these candidates, *manA* (Unigene186) had the lowest identity to its homolog in the dataset (66% to 67%). Relatively lower identity of *UXE* (Unigene 9227) to its homolog was also obtained, but the value was larger than 79% (*V*. *vinifera*, XP_002264946). For following candidates: *manB* (Unigene11590), *UGPase* (Unigene 16117), *RHM* (Unigene 7955), *UER* (Unigene 12223), and *UGlcAE* (Unigene 15345), their identities to the corresponding homolog ranged from 81% to 93%. *UGDH* (CL5269.Contig 2) and *AXS* (Unigene 14947) shared rather higher identities (equal or larger than 90%) to their homologs available in the public dataset. The detailed results of the alignment were listed in the S7 Table. The analysis primarily confirmed that the selected potential unigenes are rather conservative and should be responsible for the synthesis of CPPs.

Among these candidate unigenes, the ones encoding UXE (UDP-arabinose 4-epimerase), UER (3,5-epimerase/4-reductase), manA (mannose-6-phosphate isomerase), and manB (phosphomannomutase) should function downstream of the predicted pathway of CPPs biosynthesis (see Fig. 8), whereas the ones encoding UGDH (UDP-glucose 6-dehydrogenase), RHM (UDP-glucose 4,6-dehydratase), and UGPase should act upstream. The predicted pathway also indicated that the unigenes responsible for UGlcAE (UDP-glucuronate 4-epimerase) and AXS (UDP-apiose/xylose synthase) should possess the same precursor and work at two branch points. Real-time PCR was carried out using equally mixed total RNA extracted from all tissues collected at flowering and boll-forming stage as a template for transcription, and PCR products were detected by 1.5% agarose gel electrophoresis. The result was presented in S3 Fig. All unigenes investigated were successfully amplified, confirming the reliability and high accuracy of the used deep sequencing technique.

**Fig 10:**
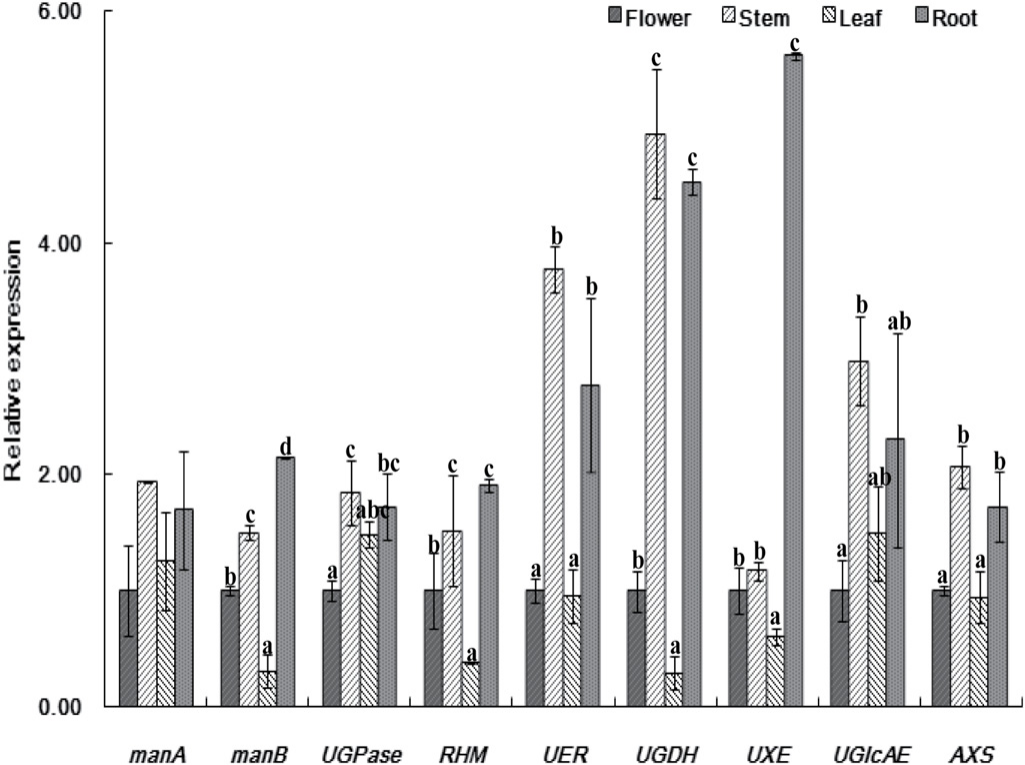
Real-time PCR analysis of candidate unigenes involved in CPP biosynthesis in *Codonopsis pilosula*. Different letter above the bar indicated significant difference in the expression of each candidate unigene in four investigated tissues. For abbreviations, please refer to Fig. 8.

The results of the relative expression of the nine candidate genes in the four tissues are presented in Fig. 10. Among these, the expression of *ManA* was relatively stable and there was no significant difference among the tissues. A similar expression pattern of *UGPase* was also observed. Relatively lower expressions in leaves and relatively higher expressions in stem and root tissues were observed for *manB*, *RHM*, *UER*, *UGDH*, *UGlcAE*, and *AXS*, indicating that the expression of these candidate genes were tissue-dependent. Comparatively, the expression of *UXE* in roots was much higher than the expression in other tissues, indicating an important role the gene might play in the biosynthesis of CPPs in the species.

**Fig 11:**
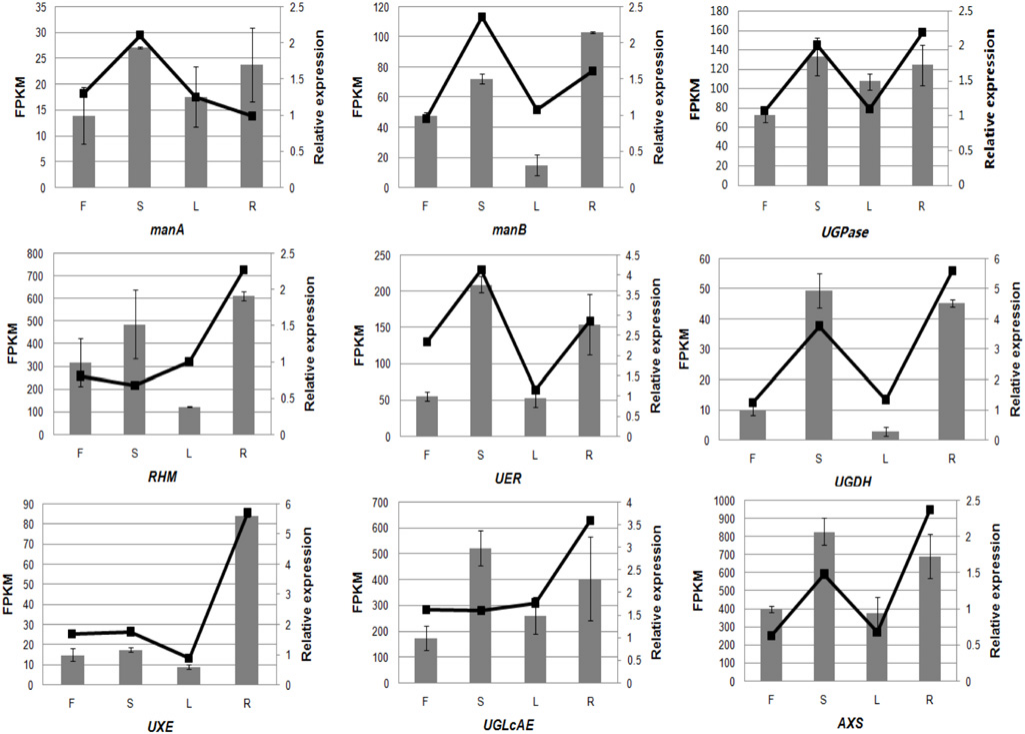
Real-time PCR validation of candidate unigenes involved in the CPP biosynthesis by RNA-seq. Relative expression dataset obtained by real-time PCR was marked with column chart. FPKM, per million mapped fragments in the transcriptome was indicated with a broken line. The left y-axis indicates gene expression levels calculated by the method of FPKM. The right y-axis indicates relative gene expression levels obtained by real time PCR, bars represent the SD (n = 3). For abbreviations, please refer to Fig. 8.

Besides real-time PCR, another dataset on the abundance of the transcripts of these unigenes was obtained by RNA-seq (S8 Table). A comparison between the two datasets was performed and was presented in Fig. 11. In the figure, the data on relative abundance of the transcripts obtained by real-time PCR were presented as a column graph, while the FPKM value of each transcript was indicated with a broken line. It revealed that there was some disagreement in the two datasets of each unigene even in the same tissue excluding *UGPase*. For example, the data from the two datasets on *manA* in roots, and *RHM* and *UGlcAE* in stems were not in accordance, indicating bias in the datasets obtained by the two methods. In general, similar patterns of gene expression in the same tissue and at the same growth stage should be observed by both techniques. In the present work, two sets of samples were collected from plants at the same growth stage but not in the same year (2012 and 2013). Inconsistent expression patterns might be caused by different environmental conditions or stress factors. The comparison between the two datasets also demonstrated that the expression of *UGPase* is relatively stable, suggesting conservation and vital role of the enzyme in the process of CPPs biosynthesis. Due to the higher sensitivity of Illumina sequencing compared to real-time PCR, further investigation is needed to understand their functions at a molecular level [51].

## Discussion

To our knowledge, this is the first *de novo* sequencing of samples from *C*. *pilosula* using Illumina HiSeq 2000 platform. The sequencing produced 91,175,044 clean reads and 45,511 unigenes after assembly and made it possible to understand the biosynthesis of CPPs at molecular level. Compared to datasets published on medicinal plants, the number of unigenes obtained from *C*. *pilosula* was lower than that obtained from *Polygonum cuspidatum* (86,418), *Dimocarpus longan* (68,925), and *Hypericum perforatum* (59,184), but was higher than that from *Siraitia grosvenorii* (43,891) [21, 24, 52, 53]. However, the average length of unigenes obtained here was 728 bp, which was longer than that from other medicinal plants, including *P*. *cuspidatum* (365 bp), *S*. *grosvenorii* (668 bp), *D*. *longan Lour*. (448 bp), and *H*. *perforatum* (422 bp). A relatively higher annotation rate (76.1%) was also observed in the present work. Notably, the number of unigenes longer than 1000-bp was 8,584, accounting for 23.75% of all unigenes, indicating higher quality of the transcriptome library and the reliability of Illumina Hiseq-2000 technology for global screening of functional genes in non-model plants, especially medicinal plants.

Using bioinformatics tools, we identified unigenes involved in CPPs biosynthesis. Annotation results suggested that sucrose is used as the primary starting material for CPPs biosynthesis. Further analysis showed relatively higher abundance of CPPs biosynthesis-related transcripts in the root and stem tissues, which might be related to the functions of the studied tissues and might be beneficial to the synthesis and accumulation of polysaccharide, the main active component in the plant. The real-time PCR analysis showed that the transcripts of genes related to the conversion of UDP-glucose to UDP-L-arabinose (branch pathway) was more abundant than those of the others, suggesting that this pathway functions as a key branch of CPPs biosynthesis at the flowering and boll-forming stage of *C*. *pilosula*. However, a previous study on *C*. *pilosula* collected during traditional harvest season showed that the content of arabinose was not the highest and was substantially lower than that of glucose and galacturonic acid [12]. NSEs are responsible for the derivatization of most NDP-sugars. The abundance of individual NSEs is associated with the activity of the enzyme and the terminal product. Consistent with a previous study, which found that xylose was an important unit in CPPs biosynthesis and that its molar ratio (15.41) was higher than that of galactose (2.87), glucose (1.92), and rhamnose (12) [54], our results showed higher abundance of *AXS* transcript in the *C*. *pilosula* library. Further studies are needed to identify the diverse molecular entities used for the biosynthesis of CPPs.

UGPase is a key enzyme that functions upstream of the biosynthesis pathway of Astragalus polysaccharides, and is responsible for the conversion of glucose-1-phosphate to UDP-Glu. Although there was a positive correlation between the activity of UGPase and the polysaccharide content in *A*. *membranaceus*, higher abundance of the corresponding transcript was not observed in our study [16]. Alignment on *UGPase* homolog from representative species in plant kingdom showed that Unigene16117 was more similar to *UGPase* from *V*. *vinifera* (Accession number: XM_002282240) than to *UGPase* from *A*. *membranaceus* (AF281081) both at nucleotide and amino acid levels. Phylogenetic analysis showed that Unigene16117 and UGPase from *Vitis vinifera* are clustered in the same subclass and that the homologues from *C*. *pilosula* and *A*. *membranaceus* are located relatively far from each other (see S2 Fig.). Therefore, the low abundance of Unigene16117 may be related to the relatively low content of CPPs in the raw materials investigated or may have been caused by divergent function of the protein. However, consistent abundance pattern of the transcript in all tissues examined across two growing seasons confirm the conservation and importance of the enzyme in the biosynthesis of CPPs. Further studies are needed to understand the relationships between enzyme activity, transcript abundance, and the content of CPPs in *C*. *pilosula*.

UDP-glucose dehydrogenase (UGDH) catalyzes the oxidation of UDP-glucose (UDP-Glc) to UDP-glucuronate (UDP-GlcA), a key sugar nucleotide involved in the biosynthesis of plant cell wall polysaccharides. Analysis on endogenous expression of a *UGDH* in *Eucalyptus grandis* (GenBank accession number: EF179384) showed that it was mainly expressed in roots, stem and bark of 6-month-old saplings, with lower expression level in leaves [55]. In *C*. *pilosula*, a relatively higher expression of *UGDH*-like gene was observed in stem and root tissues, demonstrating similar expression pattern to its homologue from *E*. *grandis*. Both works carried out in two distantly related plants also indicated the conservation of *UGDH* homology’s function in plant kingdom. L-Rhamnose (Rha) is an important constituent of CPPs, which is synthesized by the enzyme encoded by *AtRHM1* in Arabdopsis [56]. Expression studies with *AtRHM1* promoter-GUS fusion gene showed that *AtRHM1* is expressed almost ubiquitously, with stronger expression in roots and cotyledons of young seedlings and inflorescences. The most abundant transcript of *RAH1*-like unigene was detected in roots, which was consistent with *AtRHM1* in Arabidopsis. Thus, the studies on *UGDH* and *RHM* in Arabidopsis confirmed that their homology in *C*. *pilosula* should act in a similar way in the biosynthesis of CPPs, and that they should be functional genes involved in the biosynthesis of CPPs. Although UGPase, UGDH, and RHM function in parallel in separated branches of biosynthesis pathway of CPPs, the expression patterns of the latter two were more similar to each other, possessing relatively higher expression in stems and roots, indicating that the transcription of them might be regulated in a similar molecular mechanism. More work should be needed to understand the mechanism and regulation of biosynthesis and accumulation of CPPs at molecular level.

To summarize, an EST collection was created from a transcriptome library prepared from different parts of *C*. *pilosula*. The results demonstrated the usefulness of this methodology for the identification of metabolic pathways related to polysaccharide biosynthesis in medicinal plants. Our results provide understanding of the biosynthesis of CPPs at the molecular level in *C*. *pilosula* inferred by real-time PCR analysis of candidate unigenes functioning at key separate branches of biosynthesis pathway of CPPs. These findings will allow for an efficient and sustainable production of CPPs and related bioactive natural products.

## Supporting Information

S1 Fig Quantification of polysaccharides in different tissues at the flowering and boll-forming stage in *Codonopsis pilosula*. (DOC)

S2 Fig Neighbor joining (NJ) phylogenetic analysis of UGPase homologs in plants. The phylogenetic analysis was performed using MEGA 5 software. The tree was derived from nine UGPase homologs according to their amino acid sequences.
(DOC)

S3 Fig Validation of candidate unigenes involved in the biosynthesis of CPP by real-time PCR. Lanes 1 through 9 represent the amplified fragments generated via PCR. Lane 1: *manA* (162 bp); lane 2: *manB* (86 bp); lane 3: *UGPase* (192 bp); lane 4: *RHM* (111 bp); lane 5: *UER* (131 bp); lane 6: *UGDH* (193 bp); lane 7: *UXE* (97 bp); lane 8: *UGlcAE* (95 bp); lane 9: *AXS* (146 bp); lane 10: 2kb plus molecular weight marker.
(DOC)

S1 Table Statistical analysis of the efficiency of mapped reads. (XLSX)

S2 Table List of unigene numbers from *Codonopsis pilosula* assigned to KEGG reference pathway. (XLS)

S3 Table Functional annotations of unigenes involved in the biosynthesis of starch and sucrose in *Codonopsis pilosula*. (XLS)

S4 Table Functional annotations of unigenes annotated as NDP-sugar interconversion enzymes (NSEs) in *Codonopsis pilosula*. (XLSX)

S5 Table GT classification of *Codonopsis pilosula* against *Arabidopsis thaliana* GT categories. (XLS)

S6 Table Primary selection of reliable internal control for gene expression analysis in different tissues in *Codonopsis pilosula*. (XLS)

S7 Table Identity analysis of candidate unigenes related to CPP biosynthesis at encoded amino acid level. (DOC)

S8 Table FPKM values of the candidate unigenes involved in the biosynthesis of CPPs in four tissues. (XLSX)
