# Supplementary material for: Transcriptome Sequencing of Codonopsis pilosula and Identification of Candidate Genes Involved in Polysaccharide Biosynthesis
Source: PLoS One. 2015 Feb 26;10(2):e0117342. doi: 10.1371/journal.pone.0117342 (PMC4342239; doi:10.1371/journal.pone.0117342)

**Figure S1. Quantitative analysis of polysaccharide in different tissues at the flowering and boll-forming stage in *Codonopsis pilosula.***


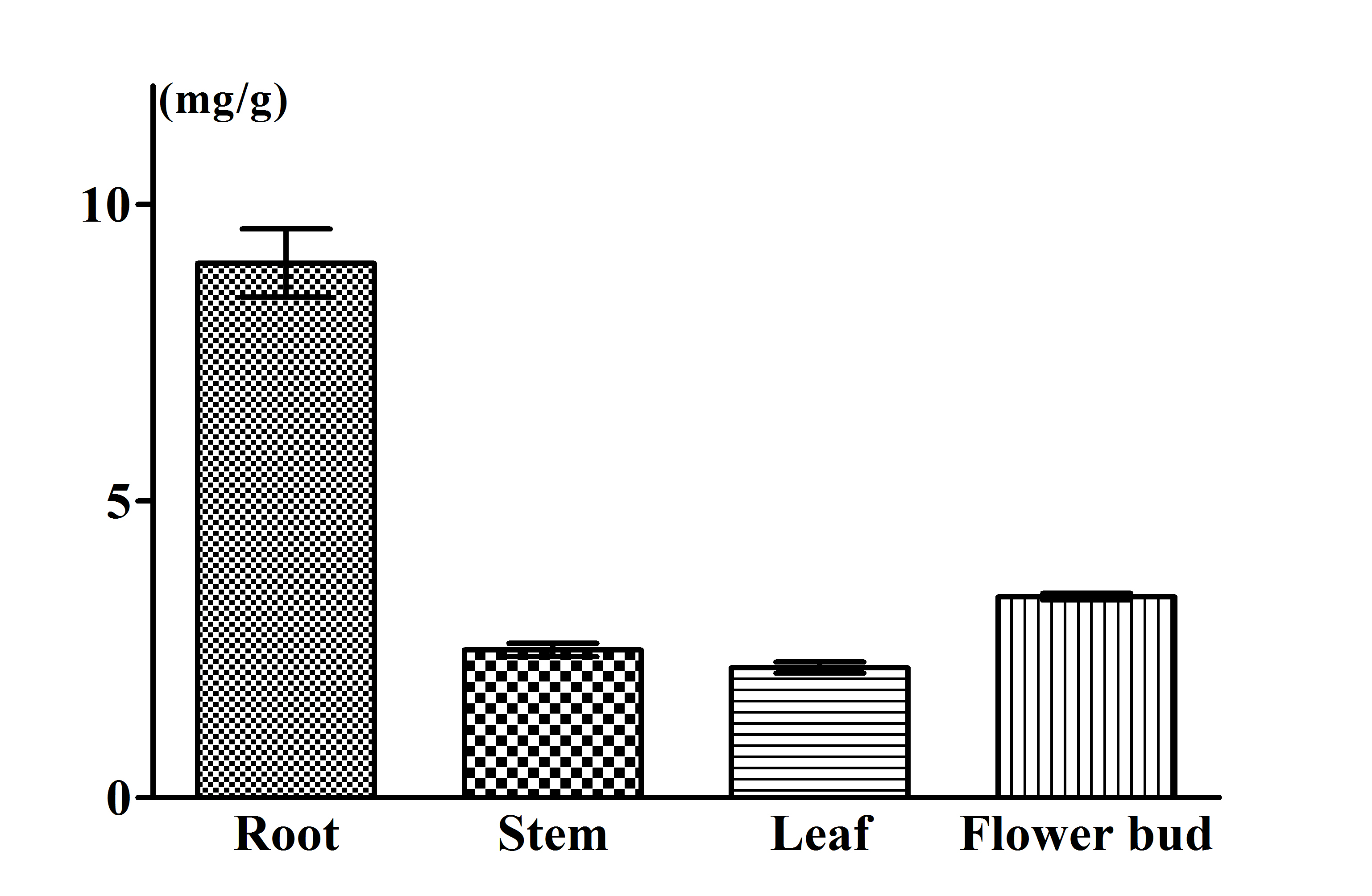

Supplement: S1 Fig — (DOC) [file pone.0117342.s001.doc]
