# Supplementary material for: Transcriptome Sequencing of Codonopsis pilosula and Identification of Candidate Genes Involved in Polysaccharide Biosynthesis
Source: PLoS One. 2015 Feb 26;10(2):e0117342. doi: 10.1371/journal.pone.0117342 (PMC4342239; doi:10.1371/journal.pone.0117342)
Supplement: S2 Fig — The phylogenetic analysis was performed using MEGA 5 software. The tree was derived from nine UGPase homologs according to their amino acid sequences. (DOC) [file pone.0117342.s002.doc]

**Figure S2.** **Neighbor-joining (NJ) phylogenetic analysis of UGPase homologs in plants.** The phylogenetic analysis was performed using MEGA 5 software. The tree was derived from nine UGPase homologs, according to their amino acid sequences.

**FvUGPase (XP 004288589) *Fragaria vesca subsp. Vesca***

**PpUGPase (AGH25528) *Prunus persica***

**RcUGPase (XP 002526594) *Ricinus communis***

**AmUGPase (AAF86501) *Astragalus membranaceus***

**GmUGPase (XP 003544964) *Glycine max***

**PtUGPase (XP 006384119) *Populus trichocarpa***

**SlUGPase (XP 004239832) *Solanum lycopersicum***

**CpUGPase (KJ470627) *Codonopsis pilosula***

**VvUGPase (XP 002282276) *Vitis vinifera***

**100**

**99**

**42**

**48**

**33**

**32**

**0.01**
