# Supplementary material for: Transcriptome Sequencing of Codonopsis pilosula and Identification of Candidate Genes Involved in Polysaccharide Biosynthesis
Source: PLoS One. 2015 Feb 26;10(2):e0117342. doi: 10.1371/journal.pone.0117342 (PMC4342239; doi:10.1371/journal.pone.0117342)
Supplement: S3 Fig — Lanes 1 through 9 represent the amplified fragments generated via PCR. Lane 1: manA (162 bp); lane 2: manB (86 bp); lane 3: UGPase (192 bp); lane 4: RHM (111 bp); lane 5: UER (131 bp); lane 6: UGDH (193 bp); lane 7: UXE (97 bp); lane 8: UGlcAE (95 bp); lane 9: AXS (146 bp); lane 10: 2kb plus molecular weight marker. (DOC) [file pone.0117342.s003.doc]

**Figure S3. Validation of candidate unigenes involved in the biosynthesis of CPP by real-time-PCR.**

Lanes 1 through 9 represent the amplified fragments generated via PCR. Lane 1: *manA* (162 bp); lane 2: *manB* (86 bp); lane 3: *UGPase* (192 bp); lane 4: *RHM* (111 bp); lane 5: *UER* (131 bp); lane 6: *UGDH* (193 bp); lane 7: *UXE* (97 bp); lane 8: *UGlcAE* (95 bp); lane 9: *AXS* (146 bp); lane 10: 2kb plus molecular weight marker.

**
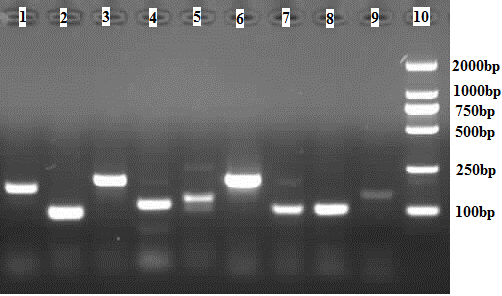
**

**(B)**

**(C)**
