# Supplementary material for: Transcriptome Sequencing of Codonopsis pilosula and Identification of Candidate Genes Involved in Polysaccharide Biosynthesis
Source: PLoS One. 2015 Feb 26;10(2):e0117342. doi: 10.1371/journal.pone.0117342 (PMC4342239; doi:10.1371/journal.pone.0117342)
Supplement: S7 Table — (DOC) [file pone.0117342.s010.doc]

**Table S7 Identity analysis of candidate unigenes related to CPP biosynthesis at encoded amino acid level.**

Sequence similarities were calculated by the Basic Local Alignment Search Tool (BLAST) (<http://blast.ncbi.nlm.nih.gov/>).

1. *manA*

From the BLAST analysis of several-deduced amino acid sequences, *manA* shared 67%, 66%, 70%, 67%, 67% and 67% identity with *Cyamopsis tetragonoloba*(AAX31279), *Theobroma cacao* (XP_007033251), *Vitis vinifera* (XP_002283926), *Morus notabilis* (EXC17063), *Solanum lycopersicum* (XP_004233489), *Solanum tuberosum* (XP_006346906).

| Gene name | Species | Accession number | Max score | Total score | Query cover | E value | Identity |
| --- | --- | --- | --- | --- | --- | --- | --- |
| *CtmanA* | *Cyamopsis tetragonoloba* | AAX31279 | 576 | 576 | 97% | 0.0 | 67% |
| *TcmanA* | *Theobroma cacao* | XP_007033251 | 582 | 582 | 97% | 0.0 | 66% |
| *VvmanA* | *Vitis vinifera* | XP_002283926.1 | 612 | 612 | 97% | 0.0 | 70% |
| *MnmanA* | *Morus notabilis* | EXC17063.1 | 605 | 605 | 100% | 0.0 | 67% |
| *SlmanA* | *Solanum lycopersicum* | XP_004233489.1 | 586 | 586 | 98% | 0.0 | 67% |
| *StmanA* | *Solanum tuberosum* | XP_006346906.1 | 582 | 582 | 98% | 0.0 | 67% |

1. *manB*

From the BLAST analysis of several-deduced amino acid sequences, *manB* shared 89%, 87%, 87%, 89%, 87%, and 87% identity with *T. cacao* (XP_007051874), *Populus trichocarpa* (XP_006375330), *Glycine max* (NP_001237668), *Camellia sinensis* (AGZ20101), *S. tuberosum* (XP_006345602), *S. lycopersicum* (NP_001233801), respectively.

| Gene name | Species | Accession number | Max score | Total score | Query cover | E value | Identity |
| --- | --- | --- | --- | --- | --- | --- | --- |
| *TcmanB* | *Theobroma cacao* | XP_007051874.1 | 441 | 441 | 99% | 4e-162 | 89% |
| *PtmanB* | *Populus trichocarpa* | XP_006375330.1 | 450 | 450 | 99% | 8e-166 | 87% |
| *GmmanB* | *Glycine max* | NP_001237668.1 | 433 | 433 | 100% | 4e-159 | 87% |
| *CsmanB* | *Camellia sinensis* | AGZ20101.1 | 438 | 438 | 99% | 6e-161 | 89% |
| *StmanB* | *Solanum tuberosum* | XP_006345602.1 | 453 | 453 | 99% | 7e-167 | 87% |
| *SlmanB* | *Solanum lycopersicum* | NP_001233801.1 | 433 | 433 | 99% | 7e-159 | 87% |

1. *UGPase*

From the BLAST analysis of several-deduced amino acid sequences, *UGPase* shared 83%, 84%, 87%, 84%, 85%, 85%, 85% and 88% identity with *Astragalus membranaceus* (AAF86501), *G. max* (XP_003544964), *P. trichocarpa* (XP_006384119), *Fragaria vesca subsp. Vesca* (XP_004288589), *Prunus persica* (AGH25528), *Ricinus communis* (XP_002526594), *S. lycopersicum* (XP_004239832), *V. vinifera* (XP_002282276) , respectively.

| Gene name | Species | Accession number | Max score | Total score | Query cover | E value | Identity |
| --- | --- | --- | --- | --- | --- | --- | --- |
| *AmUGPase* | *Astragalus membranaceus* | AAF86501.1 | 791 | 791 | 99% | 0.0 | 83% |
| *GmUGPase* | *Glycine max* | XP_003544964.1 | 803 | 803 | 100% | 0.0 | 84% |
| *PtUGPase* | *Populus trichocarpa* | XP_006384119.1 | 810 | 810 | 99% | 0.0 | 87% |
| *FvUGPase* | *Fragaria vesca subsp. Vesca* | XP_004288589.1 | 801 | 801 | 100% | 0.0 | 84% |
| *PpUGPase* | *Prunus persica* | AGH25528.1 | 751 | 751 | 93% | 0.0 | 85% |
| *RcUGPase* | *Ricinus communis* | XP_002526594.1 | 809 | 809 | 100% | 0.0 | 85% |
| *SlUGPase* | *Solanum lycopersicum* | XP_004239832.1 | 808 | 808 | 100% | 0.0 | 85% |
| *VvUGPase* | *Vitis vinifera* | XP_002282276.1 | 824 | 824 | 98% | 0.0 | 88% |

1. *RHM*

From the BLAST analysis of several-deduced amino acid sequences, *RHM* shared 84%, 83%, 81%, 85%, and 80% identity with *Zea mays* (ACG33666.1), *V. vinifera* (XP_002271970.1), *Medicago truncatula* (XP_003588651.1), *Gossypium hirsutum* (ADB24773.1), *Arabidopsis thaliana* (NP_177978.1) , respectively.

| Gene name | Species | Accession number | Max score | Total score | Query cover | E value | Identity |
| --- | --- | --- | --- | --- | --- | --- | --- |
| *ZmRHM* | *Zea mays* | ACG33666.1 | 514 | 542 | 91% | 0.0 | 84% |
| *VvRHM* | *Vitis vinifera* | XP_002271970.1 | 541 | 561 | 98% | 0.0 | 83% |
| *MtRHM* | *Medicago truncatula* | XP_003588651.1 | 505 | 526 | 91% | 7e-180 | 81% |
| *GhRHM* | *Gossypium hirsutum* | ADB24773.1 | 520 | 564 | 91% | 0.0 | 85% |
| *AtRHM* | *Arabidopsis thaliana* | NP_177978.1 | 511 | 540 | 93% | 0.0 | 80% |

E) *UER*

From the BLAST analysis of several-deduced amino acid sequences, *UER* shared 88%, 88%, 88%, 90%, and 87% identity with *V. vinifera* (XP_002282339), *Malus domestica* (XP_008348787), *Morus notabilis* (EXC17786), *G. hirsutum* (ACJ11713), *Aegilops tauschii* (EMT13548), respectively.

| Gene name | Species | Accession number | Max score | Total score | Query cover | E value | Identity |
| --- | --- | --- | --- | --- | --- | --- | --- |
| *VvUER* | *Vitis vinifera* | XP_002282339.1 | 548 | 548 | 100% | 0.0 | 88% |
| *MdUER* | *Malus domestica* | XP_008348787.1 | 544 | 544 | 100% | 0.0 | 88% |
| *MnUER* | *Morus notabilis* | EXC17786.1 | 545 | 545 | 100% | 0.0 | 88% |
| *GhUER* | *Gossypium hirsutum* | ACJ11713.1 | 557 | 557 | 100% | 0.0 | 90% |
| *AtUER* | *Aegilops tauschii* | EMT13548.1 | 543 | 543 | 100% | 0.0 | 87% |

F) *UGDH*

From the BLAST analysis of several-deduced amino acid sequences, *UGDH* shared 93%, 91%, 92%, 91%, 92% and 90% identity with *Ipomoea batatas* (AGP25694), *Eucalyptus grandis* (ABP04019), *M. notabilis* (EXB38083), *G. hirsutum* (ACJ11712), *M. truncatula* (XP_003621403), *Cinnamomum osmophloeum* (AAR84297) respectively.

| Gene name | Species | Accession number | Max score | Total score | Query cover | E value | Identity |
| --- | --- | --- | --- | --- | --- | --- | --- |
| *IbUGDH* | *Ipomoea batatas* | AGP25694.1 | 936 | 936 | 100% | 0.0 | 93% |
| *EgUGDH* | *Eucalyptus grandis* | ABP04019.1 | 919 | 919 | 100% | 0.0 | 91% |
| *MnUGDH* | *Morus notabilis* | EXB38083.1 | 919 | 919 | 100% | 0.0 | 92% |
| *GhUGDH* | *Gossypium hirsutum* | ACJ11713.1 | 920 | 920 | 99% | 0.0 | 91% |
| *MtUGDH* | *Medicago truncatula* | XP_003621403.1 | 927 | 927 | 100% | 0.0 | 92% |
| *CoUGDH* | *Cinnamomum osmophloeum* | AAR84297.1 | 919 | 919 | 100% | 0.0 | 90% |

G) *UXE*

From the BLAST analysis of several-deduced amino acid sequences, *UXE* shared 86%, 86%, 85%, 83%, 84%, and 79% identity with *M. domestica* (XP_008383358), *Prunus mume* (XP_008225692), *S. tuberosum* (XP_006356905), *A. thaliana* (NP_174350), *M. notabilis* (EXB75167), *V. vinifera* (XP_002264946), respectively.

| Gene name | Species | Accession number | Max score | Total score | Query cover | E value | Identity |
| --- | --- | --- | --- | --- | --- | --- | --- |
| *MdUXE* | *Malus domestica* | XP_008383358.1 | 746 | 746 | 98% | 0.0 | 86% |
| *PmUXE* | *Prunus mume* | XP_008225692.1 | 754 | 754 | 100% | 0.0 | 86% |
| *StUXE* | *Solanum tuberosum* | XP_006356905.1 | 746 | 746 | 99% | 0.0 | 85% |
| *AtUXE* | *Arabidopsis thaliana* | NP_174350.2 | 723 | 723 | 98% | 0.0 | 83% |
| *MnUXE* | *Morus notabilis* | EXB75167.1 | 725 | 725 | 100% | 0.0 | 84% |
| *VvUXE* | *Vitis vinifera* | XP_002264946.1 | 713 | 713 | 100% | 0.0 | 79% |

H) *UGlcAE*

From the BLAST analysis of several-deduced amino acid sequences, *UGlcAE* shared 93%, 91%, 91%, 90%, 90%, 89% and 88% identity with *M. notabilis* (EXB94890), *Cucumis melo* (XP_008465609), *P. mume* (XP_008223573), *P. trichocarpa* (XP_002324510), *G. hirsutum* (ACJ11754), *T. cacao* (XP_007013947), *A. thaliana* (NP_194773), respectively.

| Gene name | Species | Accession number | Max score | Total score | Query cover | E value | Identity |
| --- | --- | --- | --- | --- | --- | --- | --- |
| *MnUGIcAE* | *Morus notabilis* | EXB94890.1 | 837 | 837 | 99% | 0.0 | 93% |
| *CmUGIcAE* | *Cucumis melo* | XP_008465609.1 | 827 | 827 | 100% | 0.0 | 91% |
| *PmUGIcAE* | *Prunus mume* | XP_008223573.1 | 828 | 828 | 100% | 0.0 | 91% |
| *PtUGIcAE* | *Populus trichocarpa* | XP_002324510.1 | 806 | 806 | 100% | 0.0 | 90% |
| *GhUGIcAE* | *Gossypium hirsutum* | ACJ11754.1 | 818 | 818 | 99% | 0.0 | 90% |
| *TcUGIcAE* | *Theobroma cacao* | XP_007013947.1 | 815 | 815 | 100% | 0.0 | 89% |
| *AtUGIcAE* | *Arabidopsis thaliana* | NP_194773.1 | 767 | 767 | 98% | 0.0 | 88% |

I) *AXS*

From the BLAST analysis of several-deduced amino acid sequences, *AXS* shared 90%, 92%, 90%, 91%, and 90% identity with *A. thaliana* (NP_563807), *G. hirsutum* (ACJ11753), *V. vinifera* (AEP17006), *M. truncatula* (KEH29913), *Nicotiana benthamiana* (AAQ91380), respectively.

| Gene name | Species | Accession number | Max score | Total score | Query cover | E value | Identity |
| --- | --- | --- | --- | --- | --- | --- | --- |
| *AtAXS* | *Arabidopsis thaliana* | NP_563807.1 | 738 | 738 | 99 % | 0.0 | 90% |
| *GhAXS* | *Gossypium hirsutum* | ACJ11753.1 | 749 | 749 | 100% | 0.0 | 92% |
| *VvAXS* | *Vitis vinifera* | AEP17006.1 | 739 | 739 | 100% | 0.0 | 90% |
| *MtAXS* | *Medicago truncatula* | KEH29913.1 | 739 | 739 | 100% | 0.0 | 91% |
| *NbAXS* | *Nicotiana benthamiana* | AAQ91380.1 | 726 | 726 | 98% | 0.0 | 90% |
